# Perceived medical benefit, peer/partner influence and safety and cost to access the service: client motivators for voluntary seeking of medical male circumcision in Iganga District Eastern Uganda, a qualitative study

**DOI:** 10.11604/pamj.2013.15.117.2540

**Published:** 2013-08-05

**Authors:** Lubega Muhamadi, Musenze Ibrahim, Fred Wabwire-Mangen, Stefan Peterson, Steven J Reynolds

**Affiliations:** 1 District Health Office, Iganga District Administration, PO Box 358, Iganga, Uganda; 2 Division of Global Health, IHCAR, Department of Public Health Sciences, Karolinsika Institutet, Stockholm, Sweden; 3 Department of Epidemiology and Biostatistics, Makerere University School of Public Health, PO Box 7072, Kampala, Uganda; 4 School of Graduate Studies and Research Busoga University, PO BOX 154, Iganga Uganda; 5 School of Graduate Studies and Research Busoga University, PO BOX 154, Iganga, Uganda; 6 National Institutes of Health (NIH)/NIAID, International Centres for Excellence in Research, American Embassy, PO BOX 7007, Kampala, Uganda

**Keywords:** Motivators, safe male circumcision

## Abstract

**Introduction:**

Although voluntary medical male circumcision (VMMC) in Iganga district was launched in 2010 as part of the Uganda national strategy to prevent new HIV infections with a target of having 129,896 eligible males circumcised by 2012, only 35,000 (27%) of the anticipated target had been circumcised by mid 2012. There was paucity of information on why uptake of VMMC was low in this setting where HIV awareness is presumably high. This study sought to understand motivators for uptake of VMMC from the perspective of the clients themselves in order to advocate for feasible approaches to expanding uptake of VMMC in Iganga district and similar settings.

**Methods:**

In Iganga district, we conducted seven key informant interviews with staff who work in the VMMC clinics and twenty in-depth interviews with clients who had accepted and undergone VMMC. Ten focus-group discussions including a total of 112 participants were also conducted with clients who had undergone VMMC.

**Results:**

Motivators for uptake of VMMC in the perspective of the circumcised clients and the health care staff included: perceived medical benefit to those circumcised such as protection against acquiring HIV and other sexually transmitted diseases, peer/partner influence, sexual satisfaction and safety and cost to access the service.

**Conclusion:**

Since perceived medical benefit was a motivator for seeking VMMC, it can be used to strengthen campaigns for increasing uptake of VMMC. Peer influence could also be used in advocacy campaigns for VMMC expansion, especially using peers who have already undergone VMMC. There is need to ensure that safety and cost to access the service is affordable especially to rural poor as it was mentioned as a motivator for seeking VMMC.

## Introduction

In 2007, The World Health Organization (WHO) recommended voluntary medical male circumcision (VMMC) as part of a comprehensive HIV prevention package [1]. Since then, there has been a modest global increase in demand for male circumcision services [2].

In Uganda, the male circumcision programme was launched in September 2010, targeting 4,200,000 (80 percent) of uncircumcised men by 2015. By March 2012, however, only a total of 380,000 men (9.0%) had actually been circumcised under the programme [3].

In Iganga district eastern Uganda, VMMC was launched in 2010. Out of the 129,896- males targeted for circumcision between 2010 and 2012 in the district, however, only 35,000 (27% have so far been circumcised creating an unmet need of 63%. Iganga district offers VMMC services at the district hospital and three other health centres (HC) level IV once every week although occasionally the services are taken nearer to the community through clinical outreach services at the lower level health centres. The VMMC services are offered at no direct costs to the clients courtesy of funding from the government and STAR-EC/JSI. Because not every unit offers the service, however, sometimes the clients have to travel long distances to access the services. [4]. According to a recent AIDS Indicator Survey in Uganda, the HIV prevalence in the region covering Iganga district is approximately 6% [5]. There are 101 health units in the district, 83 of which are owned by the Government and 18 by NGOs. District antiretroviral (ART) services are organised according to the Uganda national hierarchical referral system, where 70 of the units are health centres; 2 (HC II) offering only cotrimoxazole refills and home-based care services to PLHIV; 27 are HC III offering health testing and counselling (HTC), pre-ARV and home-based care services to PLHIV; and 3 HC IV and 1 general hospital offering routine HTC, home-based care, pre-ARV care and ART services to PLHIV. Other care providers in the district include over 200 private drugstores/private clinics located in Iganga town and several other smaller towns scattered around the district. Most of the private providers do not provide VMMC.[6]. Iganga district has a population of approximately 600.000 people, the majority of who belong to the non-circumcising Basoga ethinic tribe. The main occupation is subsistence farming.

A review of studies carried out in Africa and other low income countries shows that culture, religion, pain, adverse effects and loss of penile sensitivity among others are motivators/barriers to uptake of VMMC especially in non-circumcising populations.[7–8]. Most of the studies are, however, either quantitative in nature or have been conducted in settings with a presumably low HIV awareness. Little is known about reasons for the low uptake of VMMC in the perspective of the clients themselves from a country like Uganda where HIV awareness is presumably high [3]. The WHO has asserted that convincing men in a largely non-circumcising country to accept VMMC is an uphill task that among others requires an in-depth understanding of the socio-cultural barriers/motivators for accepting male circumcision [9]. This study therefore sought to understand the motivators for accepting VMMC from the perspective of the clients themselves in this resource-poor setting in sub-Saharan Africa with the goal of informing policy on VMMC expansion in the context of comprehensive HIV prevention programming under similar contexts.

### Objective

The study sought to understand the motivators to accepting VMMC in the perspective of the clients themselves in this resource-poor setting in sub-Saharan Africa, as a contribution to policy and strategic promotion and advocacy for expansion of VMMC in the context of comprehensive HIV prevention programming in the country.

## Methods

### Study Setting

Between July and October 2012, we conducted this study in Iganga district to understand motivators for uptake of VMMC in the district. The study was conducted at the four health centres of Iganga hospital, Busesa HC IV, Bugono HC IV, and Kiyunga HC IV. These centres were selected because they were the only ones in the district offering routine onsite VMMC. At each centre VMMC is offered once every week. Each of the four offering centres, however, also conducts VMMC outreaches to lower level health centres twice a week. The clinics are normally managed by a clinical officer (assistant physician) and two-three nurses. Over the last four years, a total of 24 staff including clinical officers (assistant physicians) and nurses from these centres have been trained to carry out VMMC in the district[6].

### Study Design

This was a qualitative study employing key informant interviews (KI), in-depth interviews (IDIs) and focus group discussions (FGDs) for data collection.

### Sample size and Sampling procedure

### Key informant interviews

Seven key informant interviews were conducted including one with the district health officer who ideally is the district focal person for HIV care and the four in charges of the VMMC clinics, one from each the four VMMC centres. Two other staff nurses who are also counsellors one from Iganga hospital and the other from Busesa HC IV were chosen as key informants by virtue of the long time they have spent at the clinics and because of the much interaction time they spend with the clients pre and post-operative.

Similarly a total of 20 IDIs were conducted with men who had come to the centres to undergo circumcision. At each centre, the IDIs were interviewed just before they underwent the circumcision. Five respondents from each of the four centres were selected, consented and interviewed. For each centre, the respondents were purposively sampled from a framework that ensured maximum variety sampling to cater for the variations in age, occupation, marital status and distance from the VMMC clinic. These clients were chosen because they were presumed to be more “knowledge rich” on the study topic in their own situations than anybody else [10].

### Focus group discussions

We also conducted ten FGDs with clients who had undergone VMMC after an approximate healing period of five weeks to generate debate and explore views on motivators for seeking VMMC. Four of the groups included clients who had undergone VMMC at Iganga general hospital, while each of the other three remaining centres contributed two groups. At each centre, the FGD respondents were purposively sampled from a framework that ensured maximum variety sampling to cater for the variations in age, occupation, marital status and distance from the VMMC clinic. Each group consisted of 6-12 participants giving us a total of 112 respondents for all the FGDs. For all the centres, each FGD was mixed with regard to background characteristics to allow a free span of discussion across the socio-demographic exposure and experience with regard to the topic of study.

### Data collection, management and analysis

For the IDIs, a guide covering individual and family background, knowledge of VMMC and its importance, motivators for seeking VMMC, sources of knowledge about VMMC and social networks and how these affected individual health-seeking behavior was used for data collection. The IDIs were conducted in the native lusoga language and each interview lasted for about 45 to 60 minutes. A similar a guide as the one used for the IDIs was used for the FGDs. The FGDs were conducted in the native lusoga language at the nearest agreed community place such as a church or school. Each FGD lasted about one hour. Interviews stopped when it was judged that point of saturation had been reached and no more new information could be retrieved.

All the data collection processes were supervised by the first author (ML) who is an indigenous public health physician and the second author (MI), who is a social scientist with experience in qualitative research. Four research assistants who come from the study area chosen on the basis of their training and experience in carrying out social research moderated and took notes for the study. The authors trained the research assistants for two days on the study aim, design and tools. Role-plays were used to prepare the research assistants for the different situations that could arise in their interaction with the informants. Experiences from the role-plays were discussed at an extra session and further methodological guidance was given.

Data analysis was done by all the authors. It was iterative, including reviews and discussions at different stages of data collection and appropriate modifications were made in the tools to address emerging issues. All the interviews were tape recorded, transcribed and translated verbatim. The units of analysis were the transcripts from the KI, FGDs and IDIs. Content analysis was used to analyse the scripts, and this entailed reading and reviewing texts of the entire interview back and forth to identify meaningful units [11]. Meaningful units explaining motivators for accepting VMMC were identified and condensed into codes, categories and themes. The researchers shared and debated the way each of them understood or coded the data until consensus was reached on the appropriate coding.

### Ethical clearance

This study was approved by the Busoga University research and ethical Review Board, the Uganda National Council for Science and Technology as well as the district authorities in Iganga. Respondents were informed about the aims of the study, their freedom to participate or withdraw at any time, confidentiality issues, and possible harm and the benefits of the study to the community. All the study participants signed consent forms before the interviews commenced.

## Results

General characteristics of the study participants (Table 1).

**Table 1:** General characteristics of in the depth interviewees (n=20)

No	Age	Sex	Education	Religion	marriage status	Occupation
1	31	M	Secondary	Protestant	Married	Peasant
2	22	M	Secondary	Protestant	Married	Peasant
3	28	M	None	Protestant	Married	Trader
4	21	M	Secondary	Catholic	Single	peasant
5	35	M	Primary	Protestant	Married	Peasant
6	33	M	Degree	Protestant	Married	Trader
7	42	M	Primary	Protestant	Married	Peasant
8	47	M	Diploma	Catholic	Married	Peasant
9	25	M	Primary	Catholic	Single	Trader
10	33	M	Primary	None	Married	Teacher
11	19	M	Secondary	Pentecost	Single	Peasant
12	34	M	None	Traditionalist	Single	Trader
13	28	M	Secondary	Catholic	Married	Peasant
14	43	M	Primary	Protestant	Widowed	Peasant
15	28	M	Primary	Protestant	Married	Trader
16	29	M	Secondary	Protestant	Married	Peasant
17	27	M	None	Protestant	Married	Trader
18	33	M	Primary	Traditionalist	Married	Peasant
19	28	M	Secondary	Catholic	Single	Teacher
20	32	M	Primary	Anglican	Married	Trader

Motivators for uptake of safe male circumcision in the perspective of the circumcised clients and the health care providers cited in this study included: perceived medical benefit, peer/partner influence, perceived sexual satisfaction and safety and cost to access the service.

### Perceived Medical benefit

From the accounts of some clients, their decision to come for VMMC was influenced by the perception that the operation would protect them from getting sexually transmitted diseases including HIV. Some clients also asserted that the operation would help them improve their penile hygiene and hence protect them from other ailments at old age such as cancers.

**Table 2:** General characteristics of FGD interviewees (Clients who underwent VMMC) n=112

Characteristic (N)	Number(N=11)	%
**Age**		
30-39	32	28.5
40-49	48	42.8
50-59	20	17.9
60-69	12	10.8
**Marital status**		
Married	46	41.1
Single	66	58.9
Education		
None	24	21.4
Primary	51	45.5
Secondary	23	20.5
Tertiary	14	12.6
**Occupation**		
Subsistence farmer	69	61.6
Trader	24	21.4
Salary/Wage ear	19	17.0
Religion		
Anglican	55	49.2
Catholic	34	30.3
Traditional	23	20.5

*“For me I heard from the radio that male circumcision is very good because it protects someone from contracting HIV and other sexually transmitted diseases so that is what encouraged me to come.”* (IDI, 35years hospital).

The perception of perceived medical benefit from VMMC was similarly echoed by some key informants and FGDs members as in the accounts below

*“Actually when we ask some clients before the operation about what motivated them to come they say it is because they have heard that male circumcision is very good because it protects them from contracting HIV, improves penile hygiene and also protects them from penile cancers at old age.”* (KII, clinical officer, health centre).

### Peer/ partner influence

Peer or partner influence was often mentioned by the clients as a driver towards coming for the services. Some respondents reported they had been influenced to come for VMMC by either their friends at work or those that they closely associated with during leisure hours who had undergone the same.

*“Actually, you know for us we have a group of five called the camp 5. We mainly do some activities like digging or other income generating activity together, so when two of our friends went for the circumcision, they came back and told us of their experience and encouraged us to go for circumcision so the remaining three of us decided to go once together for the operation so we could be uniform.”* (IDI,22 years, health centre).

Peer influence was also echoed as a motivator for male circumcision by some members of the FGDs who confessed that they had been encouraged to go for circumcision by their colleagues.

*“For me, we have a big click of guys who socialize together especially in the evenings like going for disco dancing. So one time one of our friends told us of a program where the doctors would be visiting our health centre to offer the services to improve our health. So man being young and sexually active we decided that it was important for us to go for the operation so we went. We were actually ten of us and we all went in one group. So I think friends can actually help to give stamina and encouragement to go for the operation because you are going as a group.”* (FGD informant, 33 years, health centre)

The influence of peers was also echoed by some health workers who reported that in most cases the clients they operated on were closely associated.

*“Actually many times when we take the demographics of these clients before the operation, we find that many of them are closely related either by address or some peculiar activity. They actually sometimes come in a group and want to be worked on as a group.”* (KII, nurse, health centre.

Some men also attributed their eagerness of coming for the operation as being a consequence of their partners or wives. Some respondents reported that their seemed to be a community impression amongst the female partners that having an uncircumcised man was quite obsolete in the current sex life and hence a lot of overt or convert pressures from their partners to have the circumcision done.

*“Also all over the radio and am told even some newspapers have pictures showing that having an un circumcised man is unacceptable in the current world. For example my wife one day brought a newspaper of a woman who was wondering why her partner was still uncircumcised and she also put pressure on me to do it so I went for the VMMC”* (IDI, 42 years, hospital).

The partner influence account was echoed by the key informants and the FGDs as a motivator to taking up VMMC.

*“Some clients confess that their wives not being happy about their uncircumcised status was a big factor in them deciding to come for the VMMC.”* (KII, doctor, health centre).

*“Really I think even our wives and partners. They seem to believe that it is important to have a circumcised man or boyfriend so they put the pressure on us to do so. I know of a friend whose wife went for antenatal services and when she came back she seriously urged the man to go for circumcision. It almost caused problems in the family but later when we sat we resolved it and the man went for the operation. They are living happily now.”* (FGD informant, 37 years, hospital).

### Perceived sexual satisfaction

A few informants indicated that the main reason for them going for male circumcision was because they had heard either from friends or over the mass media that the operation improved sexual satisfaction for both the man and the woman. The staff also acknowledged that they had confessions from their clients to the effect that the latter perceived circumcision to improve their sexual prowess as men let alone improving their own sexual satisfaction.

*“You know we have been hearing over the radios and also from the sex magazines that circumcised men are actually good in bed. Because this is important for my wife and for the stability of my marriage, I decided to undergo the pain because I knew the operation would make me a stronger man.”* (IDI, 35 years, hospital).

*“Sometimes when we ask them what motivates them to come they tell us it is because they have heard that circumcision will make them better men in bed either from their colleagues who have been circumcised or from the radios.”* (KII, clinical officer, hospital).

### Safety and cost

A few clients confessed that they had been encouraged to come for VMMC because they perceived the operation to be safe given that it was being performed by trained staff. Others were encouraged by the fact that they knew the services were free of direct cost since they did not have to pay any money to get the operation done.

*“Anyway for me my problem would have been the fear of bleeding to death. But since they told us that it is the doctors going to do the operation, I felt safe for my life so that is what encouraged me to come.”* (IDI, 28 years, health centre).

The issue of cost was also echoed by members of the FGDS, some of who confessed that because the program was taking services closer to the communities, this reduced the cost of accessing the services and hence the high turn up especially for outreaches.

*“By the way, bringing the services at our health centres is another factor that encourages people to go for the VMMC. You know if the camp is far then you have to get money for transport, lunch and others but here if they are near we just walk there at no cost and the food we can even get from home.”* (FGD informant, 34 years, health centre).

The phenomenon of cost was equally echoed by the staff who acknowledged that they received more clients during outreaches to the community facilities compared to the static ones at the hospitals, a fact they attributed to reductions in cost for the client to access the service.

*“I think the issue of transport is also important. For example when we go for VMMC outreaches at the community centres, we get many clients compared to when we are static here. The clients normally complain about transport, thirst and hunger coming to the hospital, which they don’t suffer when we go for outreaches.”* (KII, nurse, hospital).

## Discussion

Our findings indicate that men accepting VMMC in Iganga district eastern Uganda is not only a function of the individual will of the clients to get circumcised but a complexity of other interrelated themes such as: perceived medical benefit, peer/partner influence, perceived sexual satisfaction, safety and cost and partner influence.

### Perceived medical benefit

Perceived medical benefit was an important factor in encouraging men to go for VMMC. Perceived medical benefit such as protection against contracting sexually transmitted infections and penile hygiene have been found as drivers for accepting VMMC in Asia, latin America and other settings [12–14]. The phenomenon of perceived medical benefit as a driver for heath seeking behavior with regard to HIV prevention, care and treatment is highlighted by several similar studies [9, 15, 16].Although not exclusive on its own, the importance or anticipated benefit of an individual undergoing a certain procedure is central to health seeking behavior [17–20]. Providing sufficient information to the masses about the importance of VMMC would therefore be a very important incentive to clients as a motivator for health seeking which should be considered both in the short and long term, since it has been found to be useful in other similar settings [13, 21, 22]. The information, however, must clearly spell out that circumcision does not give 100% prevention against HIV acquisition but is a compliment to other HIV prevention strategies. This is because certain studies have established misconceptions among communities who think that VMMC is a replacement for other HIV prevention strategies such as condom use and abstinence [23, 24].

### Peer/Partner Influence

Another motivator for seeking VMMC was the influence clients got from their partners, friends or social networks that either directly or implicitly convinced them to go for VMMC. In their socio-cultural contexts, individuals are faced with a broad array of needs related not only to HIV prevention and treatment but also emotional, material, nutritional, financial, spiritual and psychosocial support. They would therefore need a lot of support to give VMMC a priority in their busy routine. Peer/partner influence has been found to motivate individuals to seek VMMC in similar settings in Kenya and other Sub-Saharan Africa settings [7, 9, 25]. Peer educators or mobilsers have similarly been found helpful in mobilizing communities for uptake of HIV and other care services in Uganda and other settings [26–28]. The importance of positive peer influence and social support as a prerequisite for appropriate HIV care has also been reported by other studies in South Africa, Zambia, Malawi, India and Brazil [9, 29–33]. Enhancing the social support expansion of VMMC will however require efforts that do not only target the individual but also their contextual social settings of change at each level of influence [34–37]

Perceived Sexual Satisfaction Some clients attributed their willingness to undergo VMMC to the perceived advantage of better sexual satisfaction to themselves and their partners. This perception has also been found as a motivator to uptake of male circumcision in studies done in South Africa [38]. The perception is, however, contrary to quantitative studies conducted in Uganda and the United States of America which established that there was no reported difference in sexual satisfaction between circumcised men and those that are not [12, 39].

### Safety and cost

Some clients confessed that the comfort of having the operation under the trained arms of a surgeon was a motivator to them accepting VMMC. VMMC under trained hands is less likely to cause associated complications such as severe bleeding and post surgical would infection [9]. Similarly taking the services closer to the communities through carefully planned outreaches enables clients access the service at an affordable financial and economic cost. Without the outreaches, clients who live far from the facilities are likely to incur costs for transport to access the service. Sometimes this may involve selling off assets to get the money for transport. Transport costs as a barrier to care seeking and treatment adherence for PLHIV has been established by similar studies in Uganda, Botswana, Tanzania, South Africa Zambia [40–42].

One way of reducing the cost and making the service even more accessible could be to accredit and equip lower level peripheral health centres and train low cadre staff so the services can reach the poorest of the poor in this context. The use of trained and supervised low cadre staff in increasing access to HIV services for the rural poor and most vulnerable in many resource-poor settings has for example shown increased uptake, high retention rates for HIV care and increased adherence to ART [43–45].The other option would be to increase the number of outreaches to the lower level health units although this approach has the burden of sustainability.

Methodological considerations We triangulated our data collection methods (KII, FGDs, IDIs). This helped us to check for consistency and contradictions inside and across the groups and interviewees. The multi-disciplinary and native research team was useful in understanding the contextual aspects relating to accepting VMMC in the perspective of the clients themselves. We feel that the content analysis employed for this study has derived appropriate in depth analysis for the purpose of the study****.

The study was basically qualitative in nature and although it gives an indepth understanding of client motivators for accepting VMMC in their own perspective, it is not representative enough to have its findings generalized to a wider population. In terms of public policy implementation, these findings are informative and can only be applied to the study area of Iganga district and only other contexts and settings judged to be similar to Iganga district. We were not also able to explore client’s perceptions and practice after circumcision on important aspects of VMMC and HIV prevention such as condom use which could have been very useful to the debate for our findings.

## Conclusion

Since perceived medical benefit was found as a motivator for seeking VMMC, there is need to strengthen campaigns for uptake of VMMC based on clearly defined medical benefits of VMMC. Information, education and communication (IEC) materials about the medical benefit of VMMC could for example be designed and distributed to the masses. The IEC materials, however, need to be precise without any ambiguity and tailored to the local languages. Peer/partner influence was a motivator for uptake of VMMC. Designing the program based on community mobilization using the peers, especially those who have already undergone VMMC could be very influential in increasing VMMC demand. Perceived sexual satisfaction is surrounded by a lot of controversies with some studies clearly indicating evidence to the contrary. In increasing campaigns for uptake of VMMC therefore, care needs to be taken in using it as a facilitating message because fronting it could actually be counterproductive. There is also need to ensure that the services are accessed by the communities at as low a cost as possible without necessarily reducing the safety of the VMMC. This could for example be through intensifying regular outreaches or equipping and training low cadre at the peripheral health centres so as to reduce the cost for the clients accessing the service.
